# Five-year Pan-European, longitudinal surveillance of *Clostridium difficile* ribotype prevalence and antimicrobial resistance: the extended *Clos*ER study

**DOI:** 10.1007/s10096-019-03708-7

**Published:** 2019-12-07

**Authors:** Jane Freeman, Jonathan Vernon, Sally Pilling, Kirsti Morris, Scott Nicolson, Sharie Shearman, Emma Clark, Jose Alejandro Palacios-Fabrega, Mark Wilcox, the Pan-European Longitudinal Surveillance of Antibiotic Resistance among Prevalent Clostridium difficile Ribotypes’ Study Group

**Affiliations:** 1 Department of Microbiology, Leeds Teaching Hospitals Trust, Leeds, UK; 2 Healthcare Associated Infections Research Group, The Leeds Institute of Medical Research, University of Leeds, Leeds, UK; 3 Astellas Pharma, Inc., Chertsey, UK

**Keywords:** *Clostridium difficile*, Ribotype prevalence, Antimicrobial susceptibility, Antimicrobial resistance, Surveillance

## Abstract

*Clostridium difficile* infection (CDI) has been primarily treated with metronidazole or vancomycin. High recurrence rates, the emergence of epidemic PCR ribotypes (RTs) and the introduction of fidaxomicin in Europe in 2011 necessitate surveillance of antimicrobial resistance and CDI epidemiology. The *Clos*ER study monitored antimicrobial susceptibility and geographical distribution of *C. difficile* RTs pre- and post-fidaxomicin introduction. From 2011 to 2016, 28 European countries submitted isolates or faecal samples for determination of PCR ribotype, toxin status and minimal inhibitory concentrations (MICs) of metronidazole, vancomycin, rifampicin, fidaxomicin, moxifloxacin, clindamycin, imipenem, chloramphenicol and tigecycline. RT diversity scores for each country were calculated and mean MIC results used to generate cumulative resistant scores (CRSs) for each isolate and country. From 40 sites, 3499 isolates were analysed, of which 95% (3338/3499) were toxin positive. The most common of the 264 RTs isolated was RT027 (mean prevalence 11.4%); however, RT prevalence varied greatly between countries and between years. The fidaxomicin geometric mean MIC for years 1–5 was 0.04 mg/L; only one fidaxomicin-resistant isolate (RT344) was submitted (MIC ≥ 4 mg/L). Metronidazole and vancomycin geometric mean MICs were 0.46 mg/L and 0.70 mg/L, respectively. Of prevalent RTs, RT027, RT017 and RT012 demonstrated resistance or reduced susceptibility to multiple antimicrobials. RT diversity was inversely correlated with mean CRS for individual countries (Pearson coefficient r = − 0.57). Overall, *C. difficile* RT prevalence remained stable in 2011–2016. Fidaxomicin susceptibility, including in RT027, was maintained post-introduction. Reduced ribotype diversity in individual countries was associated with increased antimicrobial resistance.

**Electronic supplementary material:**

The online version of this article (10.1007/s10096-019-03708-7) contains supplementary material, which is available to authorized users.

## Introduction

*Clostridium difficile* infection (CDI) represents a major healthcare burden in the developed world [18]. Metronidazole and vancomycin have been the mainstays of CDI treatment in recent decades [24]; however, high recurrence rates and reports of reduced susceptibility to metronidazole among epidemic *C. difficile* PCR ribotypes (RTs) have highlighted the need for new agents [1, 24]. Fidaxomicin is a macrocyclic antibiotic with low MICs against *C. difficile*, approved by the EMA in 2011 for the treatment of CDI in adults [7]. In two phase 3, double-blind, randomized, parallel-group trials, it demonstrated non-inferiority in initial cure of CDI and lower rates of recurrence, compared with oral vancomycin [5, 15]. Fidaxomicin is also associated with greater preservation of the intestinal microbiota compared with vancomycin [14].

The aims of the 5-year *Clos*ER study (2011–2016) were to identify and monitor the longitudinal antimicrobial susceptibility of *C. difficile* clinical isolates, including those previously implicated in selection pressure, to establish a comprehensive susceptibility database baseline for ongoing surveillance and to provide data on the geographical distribution of clinical *C. difficile* strain types across Europe.

## Methods

*Clos*ER was a Pan-European, multicentre, in vitro surveillance study, planned to provide data for 1 year prior to the introduction of fidaxomicin (July 2011–June 2012) and 2 years post-introduction (2012–2014). It was subsequently extended for a further 2 years (2014–2016). Participating centres were mostly national or regional *C. difficile* referral laboratories selected using the European Study Group on *Clostridium difficile* (ESGCD) network and with ESGCD approval. The number of sites approached per country was based on population (1 site per 15 million population) or reported incidence of CDI (≥ 2 sites for countries with > 20 cases per 10,000 patient days per hospital). Fifty-one sites from 28 European countries were asked to participate. Criteria for site inclusion were active sampling and testing for CDI, sufficient numbers of clinical CDI cases to reach a target of 25 de-duplicated cases during the 6-month collection period and a willingness to submit the required number of samples over the 3 years. The 40 sites that contributed samples during years 1–3 were contacted to request their participation in years 4 and 5 of the study. Of these, 28 sites agreed to participate in the extended study, three sites formally ended their participation after year 3, and nine sites were unresponsive.

Isolates or faecal samples were submitted to a central laboratory (Leeds, UK) for PCR ribotyping, determination of toxin status and assessment of susceptibility to metronidazole, vancomycin, rifampicin, fidaxomicin, moxifloxacin, clindamycin, imipenem, chloramphenicol and tigecycline, using methods described previously [9, 10] (Online Resource 1). Participating sites were asked to provide the following demographic data to accompany each sample: age, gender, history of CDI in the previous 6 months, healthcare or community CDI episode, and antimicrobial administration 1 month prior to the episode.

RT diversity and Cumulative Resistance Scores were calculated each year for individual countries (Online Resource 1).

## Results

### Submissions

Across the 5 years of the study, a total of 3656 faecal samples or *C. difficile* isolates were submitted, yielding 3499 isolates of which 95% (*n* = 3338) were toxin positive (Table 1). Only six countries participated in all years of the study: Czech Republic, France, Germany, Ireland, Spain and the UK. Preliminary data for year 1 and complete data for years 1–3 have been published previously [9, 10].

**Table 1:** Numbers of participating sites and submissions received over years 1-5 of the *Clos*ER study

Year	Number of participating sites	Complete demographic data, *n*/*N* (%)	Antimicrobial treatment data, *n*/*N* (%)		Submissions received	*C. difficile*-positive isolates	Toxin-positive isolates, *n*/*N* (%)
			Datasets	Complete datasets			
1	39	7/39 (17.9)	14/39 (35.9)	3/39 (7.7)	978	944	897/944 (95.0)
2	40	6/40 (15.0)	16/40 (40.0)	7/40 (17.5)	1003	948	910/948 (96.0)
3	33	7/33 (21.2)	19/33 (57.6)	9/33 (27.3)	846	808	770/808 (95.3)
4	23	2/23 (8.7)	10/23 (43.5)	1/23 (4.3)	575	560	529/560 (94.5)
5	7	1/7 (14.3)	3/7 (42.9)	0/7 (0)	254	239	232/239 (97.1)
Total	–	–	–	–	3656	3499	3338/3499 (95.4)

Fifty-one sites in total were initially contacted. Submissions include both isolates and faecal samples

In any given year, less than one-quarter of sites submitted complete demographic datasets (Table 1). Prior occurrence of CDI was indicated for 418 (19.4%) of the 2154 samples for which information was available. There was a degree of variation between RTs with regard to prior occurrence of CDI (Table 2); however, these data should be interpreted with caution due to the lack of information for the majority of samples. Complete antimicrobial treatment data was received from < 30% of sites in each year of the study; analysis was, therefore, not performed on these data due to the potential for bias in the use of incomplete information.

**Table 2:** Proportions of the most prevalent ribotypes (*n* > 70) isolated over years 1–5 of the *Clos*ER study, by gender, age group, source and previous CDI history

Ribotype	Gender, % (*N* = 2577)		Median age (years)	Age in years, % (*N* = 2460)						Source, % (*N* = 2269)		Prior CDI, % (*N* = 2154)	
	Male	Female		< 1	1–18	19–44	45–64	65–84	≥ 85	Communityacquired	Hospitalacquired	No	Yes
027	46.56	53.44	77	0.00	0.43	3.85	14.96	58.12	22.65	20.4	72.9	65.1	26.0
014	41.18	58.82	71	1.44	2.40	9.62	20.67	44.71	21.15	23.6	67.0	67.9	23.9
001	47.00	53.00	73	0.97	1.94	9.71	20.87	53.40	13.11	9.9	79.7	79.3	12.4
078	49.69	50.31	73	0.42	6.28	14.64	33.89	14.64	30.13	24.3	70.3	73.1	18.6
002	46.73	53.27	68	1.77	2.65	15.04	20.35	50.44	9.73	24.7	66.3	75.0	14.8
020	49.59	50.41	69	3.31	7.44	14.05	16.53	43.80	14.88	17.5	76.3	70.3	22.5
005	46.75	43.25	72	0.87	2.61	24.35	32.17	30.43	9.57	22.1	75.0	70.6	17.6
126	57.45	42.55	73	1.27	1.27	8.86	35.44	31.65	21.52	14.1	83.5	73.3	23.3
015	46.75	44.87	70	9.20	5.75	16.09	20.69	28.74	19.54	31.9	62.3	84.5	12.7
All isolates	46.91	53.08	71	1.42	9.75	7.44	15.01	30.87	35.52	20.4	72.9	73.8	19.5

### PCR ribotype prevalence and distribution

**Fig. 1:**
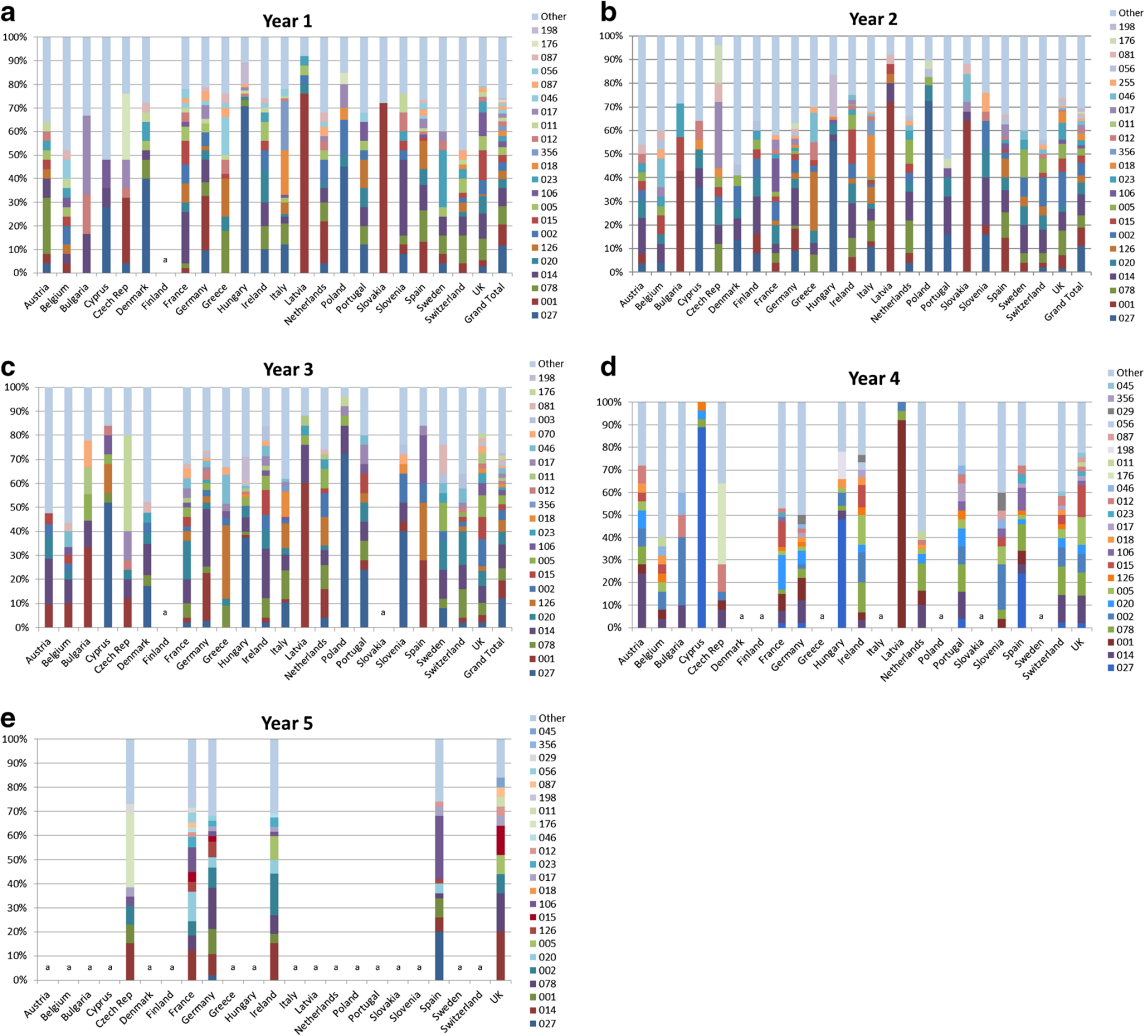
Percentage prevalence by country of *C. difficile* PCR ribotypes in **a** year 1, **b** year 2, **c** year 3, **d** year 4 and **e** year 5 of the *Clos*ER study. ^a^No submissions received from this country. Other = all other ribotypes with prevalence < 1%

Across years 1, 2, 3, 4 and 5 of the study, there were 114, 144, 120, 107 and 66 respective known PCR RTs isolated, making a total of 264 distinct RTs. The most commonly isolated RTs (prevalence ≥ 1%) in all years are listed in Table 3. RT prevalence and diversity scores varied markedly between countries and between each year of the study (Fig. 1a–e).

**Table 3:** Percentage prevalence (> 1%) of *C. difficile* PCR ribotypes in years 1–5 of the *Clos*ER study

Year 1			Year 2			Year 3			Year 4			Year 5			Total		
RT	*n*	Prevalence, %	RT	*n*	Prevalence, %	RT	*n*	Prevalence, %	RT	*n*	Prevalence, %	RT	*n*	Prevalence, %	RT	*n*	Prevalence, %
027	115	12.2	027	112	11.8	027	101	12.6	027	65	11.3	014	30	11.8	027	404	11.4
001	86	9.1	014	89	9.4	014	85	10.6	001	43	7.5	106	21	8.3	014	321	9.1
078	76	8.1	001	77	8.1	001	63	7.8	014	43	7.5	002	20	7.9	001	283	8.0
014	74	7.8	002	53	5.6	020	44	5.5	078	39	6.8	078	20	7.9	078	231	6.5
020	38	4.0	078	53	5.6	078	43	5.4	002	32	5.6	001	13	5.1	002	175	4.9
126	35	3.7	020	49	5.2	126	41	5.1	020	24	4.2	020	13	5.1	020	168	4.7
002	34	3.6	005	31	3.3	002	35	4.4	005	22	3.8	027	11	4.3	005	122	3.4
015	32	3.4	015	31	3.3	005	31	3.9	015	21	3.7	176	8	3.1	126	121	3.4
005	31	3.3	126	31	3.3	015	22	2.7	012	15	2.6	005	7	2.8	015	112	3.2
106	24	2.5	018	28	3.0	046	19	2.4	039	11	1.9	015	6	2.4	106	85	2.4
023	23	2.4	023	20	2.1	106	18	2.2	003	10	1.7	017	6	2.4	018	69	1.9
018	21	2.2	017	18	1.9	017	15	1.9	070	9	1.6	126	6	2.4	017	62	1.7
356	21	2.2	046	17	1.8	176	13	1.6	106	9	1.6	023	5	2.0	023	61	1.7
012	19	2.0	012	14	1.5	018	12	1.5	176	9	1.6	050	5	2.0	012	60	1.7
011	16	1.7	198	14	1.5	003	11	1.4	018	8	1.4	081	5	2.0	046	58	1.6
017	16	1.7	106	13	1.4	010	11	1.4	126	8	1.4	070	4	1.6	176	47	1.3
046	15	1.6	056	11	1.2	081	11	1.4	017	7	1.2	012	3	1.2	011	40	1.1
087	15	1.6	029	10	1.1	011	10	1.2	043	7	1.2	013	3	1.2	081	39	1.1
056	11	1.2	039	10	1.1	023	10	1.2	010	6	1.0	056	3	1.2	003	35	1.0
Other	241	25.5	081	10	1.1	070	10	1.2	013	6	1.0	154	3	1.2	070	35	1.0
			Other	257	27.1	012	9	1.1	054	6	1.0	Other	58	22.8	198	35	1.0
						198	8	1.0	198	6	1.0				Other	960	27.3
						Other	180	22.4	Other	160	27.9

#### Ribotype prevalence and distribution

RT prevalence results for years 1–3 have been described in detail previously [9, 10]. RT027 was the most commonly isolated RT at a mean prevalence of 11.4% across years 1–5 (Table 3). RT027 was highly prevalent in Poland for all years that submissions were received (years 1–3) and in Hungary in year 1. In Cyprus, its prevalence increased each year from 28% in year 1 to 89% in year 4 (no submissions were received from Cyprus in year 5). For countries that provided samples in all 5 years of the study, although fluctuations were apparent, the most prevalent RTs remained broadly consistent between 2011 and 2016 (Online Resource 2).

#### Ribotype diversity

**Fig. 2:**
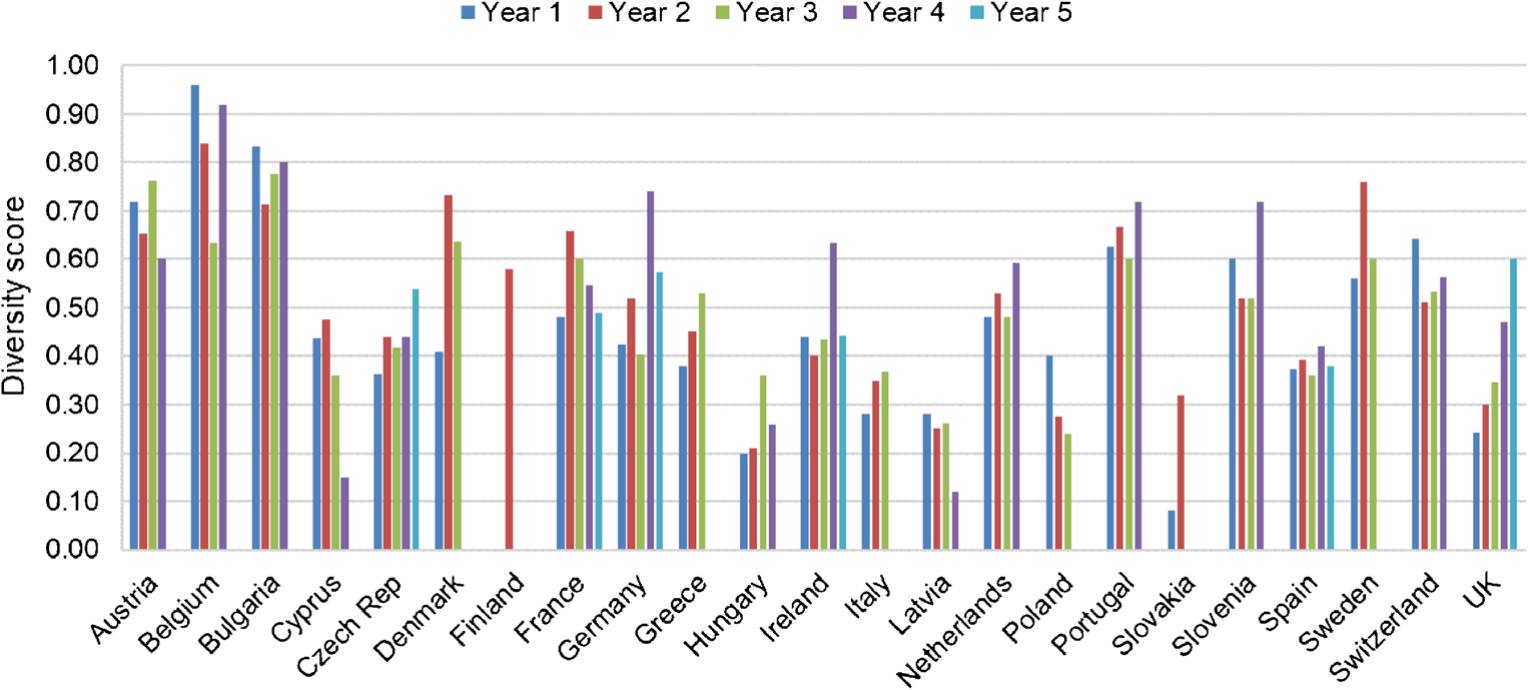
Ribotype diversity by country during years 1–5 of the *Clos*ER study

RT diversity varied between countries and between years (Fig. 2). The highest overall RT diversity was seen in Belgium (RT diversity scores 0.96, 0.84 and 0.92 for years 1, 2 and 4, respectively). Although high scores were also found for Bulgaria, it was omitted from this analysis due to the very low sample numbers submitted each year.

### Antimicrobial susceptibility

#### Fidaxomicin

Almost all isolates from years 1 to 5 were susceptible to ≤ 1 mg/L fidaxomicin (Online Resource 3; Online Resource 4; Online Resource 5), with an overall geometric mean MIC of 0.04 mg/L across all years of the study. A single fidaxomicin-resistant isolate (MIC ≥ 4 mg/L) was obtained in France in year 5 and found to be RT344. All other RT344 isolates (*n* = 5) were susceptible to fidaxomicin (MIC 0.004–0.5 mg/L) (Online Resource 6).

Fidaxomicin MICs were highest for RT198 at 0.04–0.10 mg/L over years 1–4. No RT198 was isolated in year 5. Fidaxomicin MICs for RT027 isolates varied between 0.02 and 0.08 mg/L over years 1–5. RT017, which was associated with the most isolates with multiple antimicrobial resistance in years 1, 2, 3 and 5 (see below), had MICs of 0.01–0.05 mg/L and showed no sign of reduced susceptibility to fidaxomicin. Similarly, RT012 had the most multiple antimicrobial-resistant isolates in year 4 but showed no sign of reduced susceptibility to fidaxomicin (MIC 0.03–0.05 mg/L). There was also no evidence of reduced susceptibility among newly emergent RTs with multiple antimicrobial resistance, such as RT356 and RT018, which are closely related RTs with MICs of 0.03–0.05 mg/L over the years that these were isolated.

#### Metronidazole and vancomycin

Metronidazole and vancomycin were highly active with very little variation in sensitive, intermediate and resistant isolates over years 1–4. Geometric mean MICs for years 1–5 were 0.46 mg/L and 0.70 mg/L for metronidazole and vancomycin, respectively. Reduced metronidazole susceptibility was mainly observed in RT027 and RT198 (Online Resource 4). Although vancomycin MICs above the geometric mean were observed in RT018 (years 1 and 4) and RT356 (years 1 and 3) from Italy, geometric mean vancomycin MICs for these RTs were similar to those for all isolates in other years (Online Resource 4). Geometric mean vancomycin MICs also increased in RT126 from 0.66 mg/L in year 1 to 1.00 mg/L in Year 5.

#### Rifampicin

Numbers of isolates resistant to rifampicin decreased slightly over the course of the study from 13.5% in year 1 to 10.2–11.8% in years 4–5 (Online Resource 3). The more prevalent RTs 027, 198, 018, 356, 017 and 176 had notably high proportions of rifampicin resistance.

#### Moxifloxacin and clindamycin

Resistance to both moxifloxacin and clindamycin was common and evident in all participating countries but varied between years and between countries (Online Resource 3; Online Resource 4). For example, clindamycin resistance in the Czech Republic fluctuated between 24 and 63% during the study.

#### Imipenem, chloramphenicol and tigecycline

The majority of isolates in all years were susceptible to imipenem, and the highest geometric mean MICs were found for RT176 (10.37 mg/L, year 5) and RT017 (10.56 mg/L, year 3) (Online Resource 4). The majority of isolates were susceptible to chloramphenicol, but higher geometric mean MICs were observed in RT001 and RT017. Reduced susceptibility to tigecycline (MIC > 0.25 mg/L) was also scarce; however, geometric mean MICs were marginally elevated in RT012.

### Multiple antimicrobial resistance

Of the prevalent RTs, RT027 consistently demonstrated resistance or reduced susceptibility to metronidazole, rifampicin, moxifloxacin and imipenem, while RT001 had consistently elevated geometric mean moxifloxacin, clindamycin and chloramphenicol MICs. Other prevalent RTs showed reduced susceptibility to multiple antimicrobials. Some RT017 isolates had elevated geometric mean rifampicin, moxifloxacin and clindamycin MICs in years 1 and 4 and elevated imipenem and chloramphenicol MICs in years 2, 3 and 5. RT017 in years 1–3 and year 5, plus RT012 in year 4, demonstrated resistance to the broadest range of antimicrobials tested.

### Antimicrobial susceptibility by country

Of the countries that submitted data for all 5 years of the study, Ireland and the UK showed generally reducing CRS over the first 4 years of the study, increasing slightly in year 5 (year 1: Ireland 2.26, UK 2.16; year 4: Ireland 0.87, UK 0.49; year 5: Ireland 1.22, UK 1.25) (Online Resource 7). When all countries included in the study were analysed, there was a significant inverse correlation between RT diversity and mean CRS for individual countries (Pearson coefficient *r* = − 0.57; correlation significance *p* = 0.004) (Online Resource 8). This indicated lower antimicrobial resistance levels in countries with a greater *C. difficile* RT diversity.

## Discussion

To date, this is the largest Pan-European study of *C. difficile* RT prevalence and antimicrobial resistance. Almost 3500 isolates were received, yielding 264 distinct RTs. Over the first 4 years of the study, the prevalence of the 10 most common RTs remained stable and the changes observed in year 5 should be interpreted with caution due to a substantially reduced number of submissions and participating countries. The most prevalent RTs found in our study correspond closely to those previously reported in 2011 [3] and 2016 [6], indicating overall stability in RT prevalence over time. Some fluctuation in relative RT prevalence between years and between countries was observed, as expected due to endemic and epidemic spread of *C. difficile* [8]. Previously described epidemic or highly prevalent RTs, such as 014, 027, 001 and 078, remained highly prevalent in this study. RT005, RT087 and RT356 were more prevalent in year 1 than previously observed [3], but of these, only RT005 remained highly prevalent throughout the study.

Fidaxomicin MICs remained consistently low throughout the 5 years of the study, and there was no evidence of a reduction in susceptibility following its introduction in 2011. This is consistent with an earlier antimicrobial susceptibility survey of isolates from two phase III studies of 1164 patients that reported fidaxomicin MIC90s of 0.25 mg/L [11]. The same survey identified a single strain of *C. difficile* from a patient with recurrence with a fidaxomicin MIC of 16 mg/L; however, the relatedness of the pre- and post-treatment strains was not determined [11], and the association of resistance with drug exposure cannot be made definitively [21]. Schwanbeck et al. described fidaxomicin resistance (MIC > 64 mg/L), associated with a V1143D mutation in rpoB, in a single clinical *C. difficile* isolate of 50 isolates tested [19]; however, the fitness burden imposed by the mutation was higher when generated in vitro [13, 19] than observed in the clinical isolate [19]. In the light of high fidaxomicin gut concentrations, the significance of this is unclear but highlights the need for further monitoring. We report a single fidaxomicin-resistant isolate of RT344 (MIC ≥ 4 mg/L), isolated in year 5 of the study; this isolate was also resistant to moxifloxacin, clindamycin and imipenem, but sensitive to all other antimicrobials tested. All other RT344 isolates submitted were susceptible to fidaxomicin.

RT027, the most prevalent RT in the *Clos*ER study, has previously been associated with multiple antimicrobial resistance [17] and reduced susceptibility to fidaxomicin compared with other PCR ribotypes (MIC90s 0.5 mg/L versus ≤ 0.25 mg/L, respectively) [11]. However, no such association was found in the *Clos*ER study. The geometric mean fidaxomicin MIC for the RT027 isolates submitted to this study (0.04–0.08 mg/L) was below the susceptibility breakpoint. Likewise, RT017, 012, 018 and 356 showed resistance to multiple antimicrobials in this study but were not associated with higher fidaxomicin MICs. Moreover, the clinical significance of RT-specific variations in fidaxomicin susceptibility is questionable, particularly given the high concentrations of fidaxomicin (> 1000 μg/g) attained in the gut [20].

While susceptibility to fidaxomicin remained stable over years 1–5, susceptibility to metronidazole and vancomycin increased. This could be attributed to a reduction in metronidazole and vancomycin use and/or greater strain diversity. The present study confirmed previously reported associations between prevalent RTs, such as RT027 and RT001, and resistance to moxifloxacin, clindamycin and chloramphenicol [2, 16, 22]. However, there were examples of these RTs from many countries showing almost full susceptibility to all agents. Imipenem resistance is not well-documented for *C. difficile*, but we found evidence of both intermediate and full resistance in all years.

Fluctuations in antimicrobial susceptibility between countries and between years are reflective of the varying prevalence of RTs. Multiple antimicrobial resistance was most evident in certain epidemic RTs, such as RT027 and 001, but was also notable in RT017, RT012, emerging RT198 (exclusive to Hungary) and RT356 (exclusive to Italy). We found a consistent inverse correlation between RT diversity and mean CRS for a given country, possibly due to the introduction of mandatory reporting programmes with a subsequent increase in awareness, antimicrobial stewardship and infection control interventions reducing rates of endemic RTs.

The selection criteria for submissions stipulated 25 de-duplicated toxin-positive faecal samples or *C. difficile* isolates, with no further requirements. There may, therefore, be selection bias in the samples submitted from any location. Participating centres were mainly national or regional *C. difficile* reference facilities and, therefore, some submissions likely included outbreak strains, possibly influencing the data. The majority of submissions were isolates rather than faecal samples. The recovery rate was generally poorer from faecal samples (mean 86%; median 84%) than from isolates (mean 97%; median 100%). In years 1 and 2, three sites submitted faecal samples, with recovery rates between 64 and 100%. In years 3 and 4, two of these sites submitted isolates: for one of these sites, recovery rates increased to 100% in both years, while for the other site, recovery rates increased to 84% and 92% in years 3 and 4, respectively.

We advised sites on how to prepare isolates as spores for transport but did not obtain information on whether this advice had been followed. We experienced consistently poor *C. difficile* recovery from isolate submissions by one site during all 3 years of their participation (76%, 40% and 52%). Another site had a recovery rate of 100% in year 1, but this rate dropped to 88% and 82% for years 2 and 3, respectively, after changing to different transport conditions. It was notable that all of these were not submitted as advised in transport media, and it is possible that this contributed to the poor recovery rates. However, these sites were not the only participating locations from their country (1 of 3 and 1 of 4, respectively) and, therefore, the effect of low recovery rates was lessened somewhat. Despite these site-specific limitations, overall recovery rates were > 96% for 90%, 83%, 85% and 91% of sites in years 1, 2, 3 and 4, respectively.

There was a substantial decrease in the responsiveness of sites, and consequently the number of submissions, during years 4 and 5. Possible reasons for a lack of response included site staff resourcing issues, the extended duration of the study and the loss or retirement of named site contacts or national coordinators. Although sample transport was provided, courier transport was problematic in some countries and there was no financial incentive for sites to submit samples. In year 5, only the Czech Republic, France, Germany, Ireland, Spain and the UK submitted isolates. From the UK, only one site submitted samples in year 5, while all four sites consistently submitted samples in years 1–4. Countries with high RT027 prevalence were, therefore, not represented in year 5, skewing the data. Accompanying patient data, particularly information on antimicrobial treatment, was often missing.

## Conclusions

Overall ribotype prevalence across Europe remained stable between 2011 and 2016, and a lack of ribotype diversity in an individual country was associated with greater antimicrobial resistance. There was no evidence of reduced susceptibility to fidaxomicin following its introduction in 2011. The identification of emerging and highly antimicrobial-resistant *C. difficile* PCR ribotypes highlights the importance of continued surveillance.

## Electronic supplementary material

ESM 1 (PDF 825 kb)
