# Supplementary material for: Five-year Pan-European, longitudinal surveillance of Clostridium difficile ribotype prevalence and antimicrobial resistance: the extended ClosER study
Source: Eur J Clin Microbiol Infect Dis. 2019 Dec 7;39(1):169–77. doi: 10.1007/s10096-019-03708-7 (PMC6962284; doi:10.1007/s10096-019-03708-7)

SUPPLEMENTARY INFORMATION

Article title: Five-year pan-European, longitudinal surveillance of *Clostridium difficile* ribotype prevalence and antimicrobial resistance: the extended *Clos*ER study

Target journal: *European Journal Clinical Microbiology & Infectious Diseases*

Authors: Jane Freeman[1,2], Jonathan Vernon[2], Sally Pilling[2], Kirsti Morris[1], Scott Nicolson[2], Sharie Shearman[2], Emma Clark[2], Jose Alejandro Palacios-Fabrega[3], Mark Wilcox[1,2] and the Pan-European Longitudinal Surveillance of Antibiotic Resistance among Prevalent *Clostridium difficile* Ribotypes' Study Group

*__Corresponding author__:
Dr Jane Freeman
Department of Microbiology
Old Medical School
Leeds General Infirmary
Leeds
LS1 3EX
West Yorkshire
UK
Telephone: 0113 3928663
Email: jane.freeman4@nhs.net

Online Resource 1

Supplementary Methods

Culture and toxin testing

Alcohol-shocked faecal specimens or *C. difficile* isolates were inoculated on to cycloserine-cefoxitin-egg-yolk agar (LabM, Heywood, Lancashire, UK) with lysozyme and cultured anaerobically for 48 h at 37°C. Forty-eight hour anaerobic brain-heart infusion broth culture supernatants of each test isolate were added to a Vero cell culture cytotoxicity assay with *Clostridium sordellii* antitoxin (ProLab Diagnostics, Bromborough, UK) neutralisation

PCR ribotyping

PCR ribotyping using capillary electrophoresis was performed on each isolate by the *C. difficile* Ribotyping Network (CDRN) Reference Laboratory at Leeds Teaching Hospitals Trust, Leeds, UK, according to the method of Stubbs *et al*. [12, 23]. Ribotypes (RTs) were assigned against the UK *C. difficile* reference library at Leeds

Susceptibility testing

Minimum inhibitory concentrations (MICs) of metronidazole, vancomycin, rifampicin, chloramphenicol (Sigma, St Louis, MO, USA); moxifloxacin (Bayer, Leverkusen, Germany), clindamycin, tigecycline (Pfizer, New York, NY, USA), imipenem (MSD, Hoddesdon, UK), and fidaxomicin (Astellas, Tokyo, Japan) were determined for each isolate using a Clinical and Laboratory Standards Institute (CLSI) agar incorporation method with Wilkins–Chalgren agar, as previously described [1, 4, 9]. Susceptibility breakpoints were defined according to CLSI or European Committee on Antimicrobial Susceptibility Testing (EUCAST) recommendations. Fidaxomicin breakpoints were defined according to the manufacturer’s stated epidemiological cut-off (ECOFF) value [7].

Ribotype diversity scores

RT diversity scores for each country were calculated by dividing the total number of RTs detected, irrespective of prevalence, in that country by the number of isolates tested for that country. Numbers closest to 1 indicate the highest RT diversity and those closest to 0 correspond to the lowest RT diversity.

Antimicrobial susceptibility scores by country

For each isolate, MICs were designated susceptible (S), intermediately resistant (I) or fully resistant (R). Each result was assigned a score and a cumulative resistance score (CRS) for all of the nine antimicrobials tested was then generated for each isolate. An isolate that was fully susceptible to 4, intermediately resistant to 2, and resistant to 3 antimicrobials would therefore generate a score of 8 (0+0+0+0+1+1+2+2+2). Isolates were grouped by country and a mean CRS was generated for each country.

Statistical analyses

The relationship between RT diversity and mean CRS scores was analysed using the Pearson correlation coefficient (Fisher's Normal Transformation) in Excel Analyse-IT. Fidaxomicin MICs were compared using one-way analysis of variance for independent samples with Tukey's Honest Significant Difference test, at http://www.vassarstats.net/.

Online Resource 2

Percentage prevalence of the most common *C. difficile* PCR ribotypes for countries with submissions in all five years of the *Clos*ER study

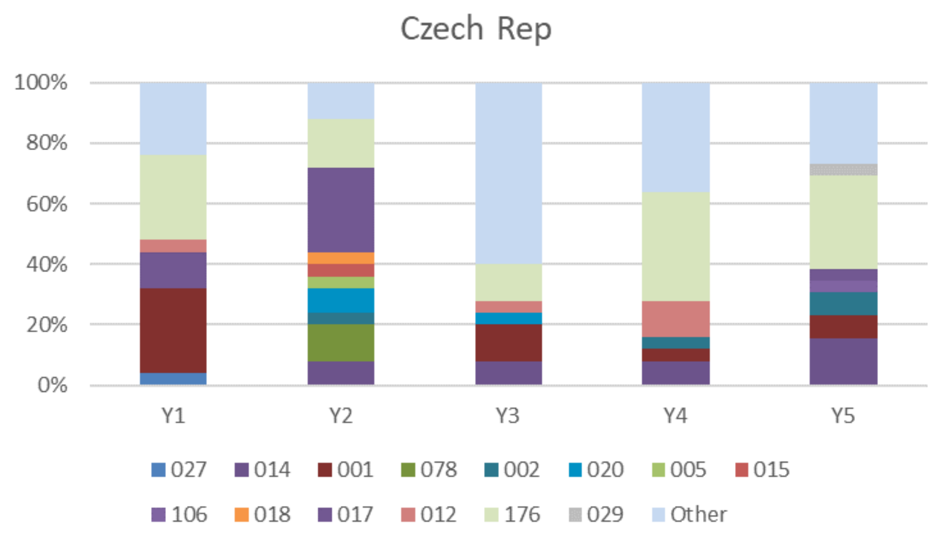

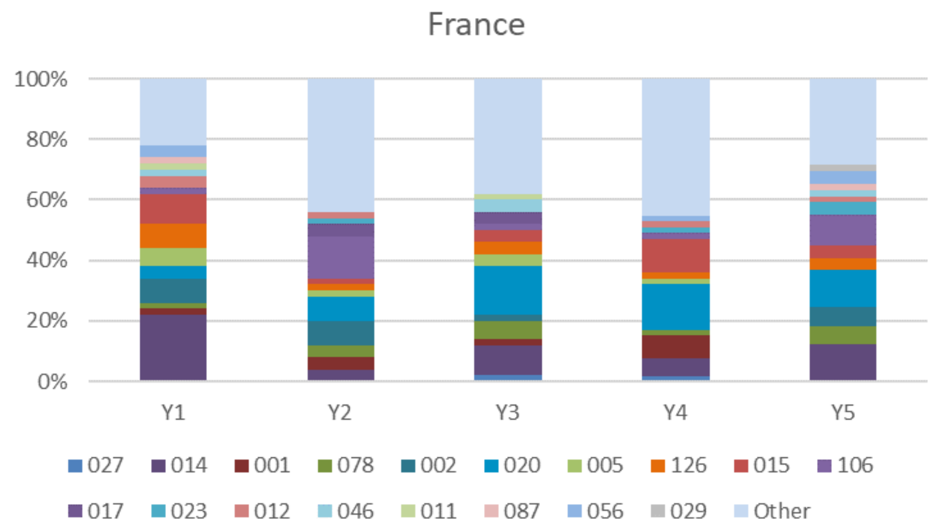

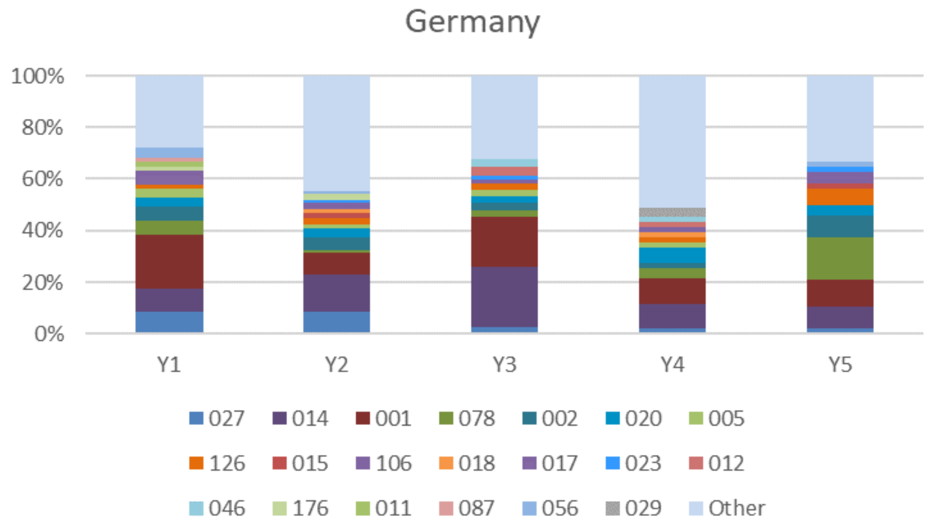
Germany
100%
80%
60%
40%
20%
0%
Y1
Y2
Y3
Y4
Y5
027 014 001 078 002 020 005
126 015 106 018 017 023 012
046 176 011 087 056 029 Other

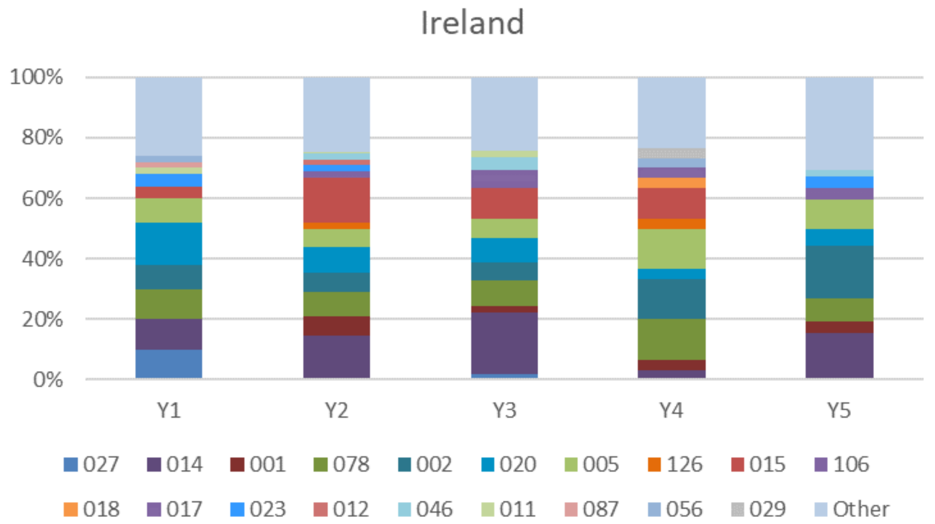
Ireland
100%
80%
60%
40%
20%
0%
Y1
Y2
Y3
Y4
Y5
027 014 001 078 002 020 005 126 015 106
018 017 023 012 046 011 087 056 029 Other

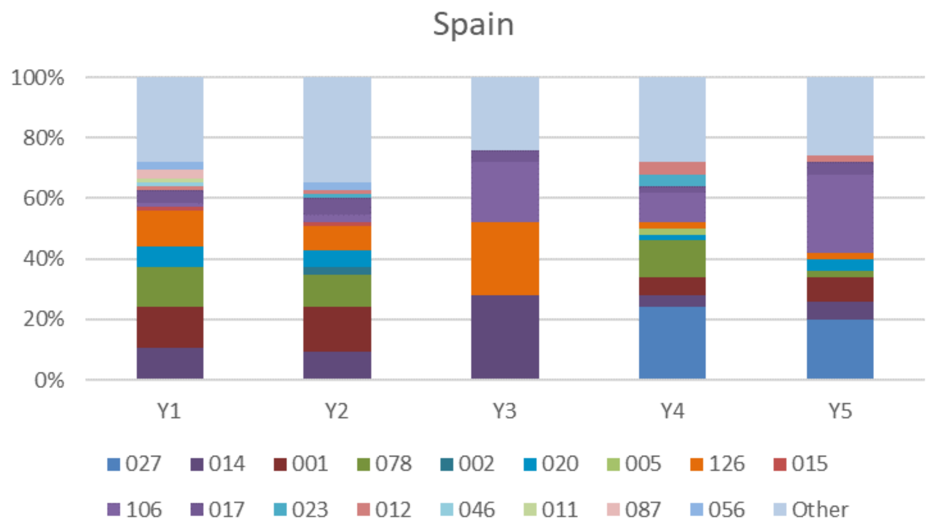
Spain
100%
80%
60%
40%
20%
0%
Y1
Y2
Y3
Y4
Y5
027 014 001 078 002 020 005 126 015
106 017 023 012 046 011 087 056 Other

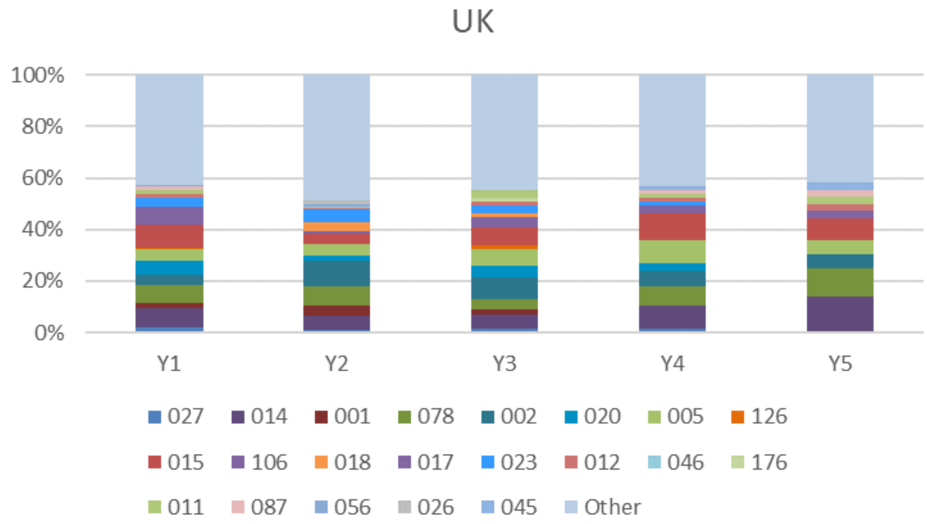

Other = all other ribotypes with prevalence <1%

Online Resource 3

Proportions of sensitive, intermediate and resistant *C. difficile* isolates from Years 1–5 of the *Clos*ER study

	**M**	**V**	**FDX**	**RIF**	**MXF**	**CLINDA**	**IMI**	**CHLOR**	**TIG**
					**Sensitive isolates, % (n/N)**				
Antimicrobial concentration, mg/L (breakpoint)	≤2	≤2	≤1	≤0.004	≤2	≤2	≤4	≤8	≤0.25
Year 1	97.9 (923/943)	96.7(912/943)	100.0 (943/943)	80.5 (759/943)	58.7 (553/942)	37.6 (354/942)	62.7 (591/943)	92.9 (876/943)	100.0 (943/943)
Year 2	98.1 (935/948)	98.8 (942/948)	100.0 (948/948)	82.1 (783/948)	64.5 (616/948	29.1 (279/948)	77.3 (739/948)	93.1 (888/948)	100.0 (948/948)
Year 3	96.9 (778/803)	99.8 (801/803)	100.0 (803/803)	86.8 (705/802)	66.0 (531/804)	18.3 (147/803)	78.1 (628/803)	91.5 (735/803)	100.0 (803/803)
Year 4	99.5 (557/560)	100.0 (560/560)	100.0 (560/560)	88.4 (494/559)	70.5 (395/560)	53.2 (305/560)	66.4 (372/560)	96.8 (541/559)	99.3 (559/560)
Year 5	100.0 (242/242)	100.0 (245/245)	99.6 (244/245)	85.3 (209/245)	67.6 (164/244)	54.5 (133/245)	60.2 (148/245)	97.6 (240/245)	100.0 (245/245)

	M	V	FDX	RIF	MXF	CLINDA	IMI	CHLOR	TIG
TOTAL	98.3 (3435/3496)	90.3 (3160/3499)	99.98 (3498/3499)	84.4 (2950/3497)	64.5 (2256/3498)	34.8 (1218/3498)	70.8 (2478/3499)	68.7 (2404/3497)	99.97 (3498/3499)
**Intermediate isolates, % (n/N)**									
Antimicrobial concentration, mg/L	4	4		0.008-16	4	4	8	16	
Year 1	2.0 (19/943)	2.4 (23/943)		6.0 (57/943)	1.8 (17/942)	12.4 (117/942)	30.1 (284/943)	3.5 (33/943)	
Year 2	1.3 (12/948)	0.6 (6/948)		3.7 (30/948)	0.9 (9/948)	13.7 (129/948)	19.7 (187/948)	2.6 (25/948)	
Year 3	2.6 (21/803)	0.1 (1/803)		1.5 (5/802)	0.5 (4/804)	17.4 (140/803)	19.7 (158/803)	5.1 (41/803)	
Year 4	0.4 (2/560)			1.4 (8/559)	0.5 (3/560)	10.357 (58/560)	31.4 (176/560)	0.9 (5/559)	
Year 5				2.9 (7/245)	0.8 (2/244)	17.1 (42/245)	35.8 (88/245)	1.2 (1/246)	
TOTAL	1.5 (54/3496)	0.86 (30/3499)		3.1 (107/3497)	1.0 (35/3498)	13.9 (486/3498)	24.7 (893/3499)	3.0 (105/3497)	

	M	V	FDX	RIF	MXF	CLINDA	IMI	CHLOR	TIG
					**Resistant isolates, % (n/N)**				
Antimicrobial concentration, mg/L	≥8	≥8	>1 (reduced susceptibility)	≥16	≥8	≥8	≥16	≥32	>0.25 (reduced susceptibility)
Year 1	0.1 (1/943)	0.8 (8/943)		13.5 (127/943)	39.5 (372/942)	49.8 (471/942)	7.2 (68/943)	3.6 (34/943)	
Year 2	0.1 (1/948)			13.7 (130/948)	34.1 (323/948)	56.6 (540/948)	2.3 (34/943)	3.7 (35/948)	
Year 3	0.5 (4/803)	0.1 (1/803)		11.6 (92/802)	33.5 (268/804)	64.3 (516/803)	2.2 (17/803)	3.4 (27/803)	
Year 4	0.2 (1/560)			10.2 (57/559)	28.9 (162/560)	35.18 (197/560)	2.1 (12/560)	2.3 (13/559)	0.2 (1/560)
Year 5			0.4 (1/245)	11.8 (29/245)	31.6 (77/244)	28.6 (70/245)	4.1 (88/245)	1.2 (3/246)	
TOTAL	0.2 (6/3496)	0.03 (9/3499)	0.02 (1/3499)	12.4 (432/3497)	40.1 (1401/3498)	51.3 (1794/3498)	6.3 (219/3499)	3.2 (112/3497)	0.03 (1/3499)

M, metronidazole; V, vancomycin; FDX, fidaxomicin; RIF, rifampicin; MXF, moxifloxacin; CLINDA, clindamycin; IMI, imipenem; CHLOR, chloramphenicol; TIG, tigecycline

Online Resource 4

MICs (mg/L) of prevalent *C. difficile* PCR ribotypes over Years 1–5 of the *Clos*ER study

Year	Ribotype	M	V	FDX	RIF	MXF	CLINDA	IMI	CHLOR	TIG
1	027	1.41	0.70	0.04	0.472	22.16	3.55	7.13	4.91	0.04
2	027	1.47	0.81	0.07	0.303	22.71	4.82	6.94	5.15	0.04
3	027	1.24	0.71	0.08	0.351	18.35	6.34	6.69	5.72	0.03
4	027	0.89	0.66	0.03	0.512	25.49	2.18	7.10	4.81	0.03
5	027	1.00	0.53	0.02	14.136	4.00	1.46	6.62	7.51	0.04
1	001	0.47	0.83	0.01	0.007	12.18	48.63	5.18	8.74	0.04
2	001	0.33	0.64	0.01	0.006	14.06	41.84	3.48	6.84	0.04
3	001	0.38	0.66	0.02	0.006	13.45	54.40	5.08	7.91	0.03
4	001	0.50	0.72	0.01	0.003	28.13	62.98	6.08	7.38	0.05
5	001	0.31	0.62	0.01	0.039	2.11	16.00	3.79	6.82	0.04
1	078	0.27	0.63	0.04	0.003	3.39	2.85	3.18	5.07	0.05
2	078	0.21	0.69	0.05	0.003	2.63	4.93	3.04	4.21	0.04
3	078	0.19	0.62	0.05	0.003	2.51	6.92	3.09	4.93	0.04
4	078	0.15	0.77	0.02	0.002	2.75	2.71	3.53	5.61	0.04

Year	Ribotype	M	V	FDX	RIF	MXF	CLINDA	IMI	CHLOR	TIG
5	078	0.17	0.72	0.02	0.002	3.10	2.15	4.63	4.63	0.04
1	014	0.27	0.65	0.06	0.002	2.41	5.15	4.31	5.87	0.05
2	014	0.25	0.60	0.06	0.003	1.95	4.83	3.16	4.90	0.04
3	014	0.22	0.63	0.06	0.003	2.00	8.20	3.77	5.97	0.04
4	014	0.18	0.59	0.04	0.002	2.07	3.27	4.07	5.33	0.04
5	014	0.17	0.52	0.02	0.002	1.91	2.39	4.51	4.51	0.03
1	020	0.31	0.73	0.07	0.002	2.63	6.43	4.80	5.66	0.05
2	020	0.27	0.64	0.06	0.002	2.07	4.06	2.96	5.09	0.05
3	020	0.22	0.65	0.06	0.002	1.68	6.94	3.42	5.75	0.04
4	020	0.23	0.56	0.03	0.002	2.18	5.19	4.62	5.82	0.04
5	020	0.18	0.50	0.03	0.002	3.23	4.69	4.45	5.22	0.04
1	126	0.23	0.66	0.04	0.003	9.37	15.69	3.62	5.38	0.06
2	126	0.23	0.60	0.05	0.003	5.79	25.40	3.10	4.59	0.05
3	126	0.17	0.62	0.04	0.003	4.58	15.47	3.05	5.52	0.04
4	126	0.15	0.74	0.02	0.002	5.38	2.21	2.69	5.38	0.04
5	126	0.20	1.00	0.02	0.003	3.56	7.13	4.00	4.49	0.06
1	002	0.24	0.65	0.05	0.002	2.13	6.80	4.00	5.89	0.04

Year	Ribotype	M	V	FDX	RIF	MXF	CLINDA	IMI	CHLOR	TIG
2	002	0.25	0.74	0.06	0.003	1.86	5.40	3.04	4.74	0.03
3	002	0.19	0.74	0.07	0.002	2.25	7.54	3.55	5.17	0.03
4	002	0.17	0.79	0.04	0.004	1.76	3.91	3.67	4.97	0.03
5	002	0.17	0.76	0.02	0.002	1.59	2.00	4.49	4.32	0.03
1	015	0.20	0.62	0.04	0.001	1.58	3.15	5.30	5.30	0.05
2	015	0.23	0.70	0.04	0.003	1.54	1.65	3.23	4.00	0.04
3	015	0.19	0.60	0.04	0.002	1.21	3.31	4.13	5.15	0.03
4	015	0.14	0.72	0.04	0.002	1.70	1.64	4.72	5.38	0.04
5	015	0.20	0.56	0.02	0.002	1.78	1.26	5.66	4.00	0.03
1	005	0.26	0.82	0.05	0.003	2.50	4.28	5.12	5.00	0.05
2	005	0.23	0.83	0.05	0.002	1.62	4.70	3.10	4.19	0.05
3	005	0.22	0.87	0.06	0.003	1.60	5.59	3.42	5.47	0.04
4	005	0.16	0.73	0.03	0.002	1.21	1.88	3.21	4.83	0.04
5	005	0.14	0.50	0.01	0.002	2.83	1.00	4.00	3.56	0.03
1	106	0.65	0.82	0.07	0.002	8.48	6.17	6.54	6.92	0.04
2	106	0.40	0.90	0.04	0.004	5.22	14.38	4.95	7.58	0.05
3	106	0.37	0.71	0.08	0.003	5.44	8.00	4.67	4.67	0.04

Year	Ribotype	M	V	FDX	RIF	MXF	CLINDA	IMI	CHLOR	TIG
4	106	0.16	0.63	0.02	0.005	8.00	1.85	4.67	4.76	0.03
5	106	0.26	0.55	0.04	0.002	4.27	4.88	5.56	5.94	0.04
1	023	0.19	0.72	0.06	0.003	1.83	0.83	3.65	6.48	0.05
2	023	0.25	0.75	0.07	0.002	1.39	1.34	3.59	4.46	0.04
3	023	0.22	0.76	0.07	0.002	1.74	1.00	3.25	5.66	0.04
4	023*	0.18	0.35	0.02	0.002	1.41	0.59	2.38	4.00	0.03
5	023	0.15	0.50	0.05	0.003	1.68	0.59	4.76	4.00	0.04
1	018	0.41	2.00	0.04	2.072	35.33	4.42	5.56	5.04	0.04
2	018	0.31	0.69	0.05	1.111	11.89	7.43	5.80	4.76	0.04
3	018	0.20	0.67	0.03	1.425	11.31	8.00	5.34	5.04	0.04
4	018	0.19	1.00	0.03	0.002	0.92	2.38	3.67	4.36	0.04
5	018	not isolated								
1	356	0.61	2.28	0.04	18.871	50.80	8.55	5.86	4.88	0.04
2	356	0.57	0.57	0.05	32.000	32.00	13.93	6.06	4.59	0.05
3	356	0.13	1.00	0.03	32.000	16.00	32.00	4.00	4.00	0.03
4	356	not isolated								
5	356	not isolated								

Year	Ribotype	M	V	FDX	RIF	MXF	CLINDA	IMI	CHLOR	TIG
1	012	0.27	0.72	0.05	0.005	2.40	51.42	5.36	9.60	0.08
2	012	0.39	0.91	0.05	0.002	1.81	55.17	3.81	6.56	0.08
3	012	0.29	0.79	0.06	0.002	2.52	69.12	4.00	11.76	0.06
4	012	0.21	0.74	0.03	0.007	2.83	43.07	4.64	9.28	0.04
5	012	0.20	0.50	0.04	0.050	1.59	6.35	1.59	3.17	0.06
1	017	0.27	0.65	0.02	0.925	18.22	43.34	5.91	7.66	0.04
2	017	0.22	0.61	0.02	1.187	13.59	39.24	9.81	14.75	0.05
3	017	0.22	0.72	0.05	0.420	9.19	50.80	10.56	14.59	0.05
4	017	0.13	0.74	0.02	0.505	9.75	11.89	4.42	6.56	0.05
5	017	0.20	0.50	0.01	0.201	7.13	57.02	8.98	8.98	0.04
1	198	1.19	0.39	0.04	0.006	5.66	0.77	7.34	4.00	0.03
2	198	1.64	0.55	0.08	0.015	23.78	12.49	7.61	4.88	0.03
3	198	1.68	0.50	0.10	0.004	24.68	4.00	8.00	8.00	0.04
4	198	2.52	0.79	0.07	0.225	28.51	3.56	7.13	5.66	0.04
5	198	not isolated								
1	176	2.18	0.65	0.02	1.009	26.91	2.00	5.66	3.08	0.04
2	176	0.77	0.65	0.05	0.848	17.45	1.41	7.34	3.67	0.03

Year	Ribotype	M	V	FDX	RIF	MXF	CLINDA	IMI	CHLOR	TIG
3	176	2.11	1.00	0.08	0.601	30.34	4.69	9.90	4.22	0.04
4	176	1.00	0.50	0.04	32.000	16.00	0.68	8.00	4.00	0.03
5	176	1.41	0.59	0.03	32.000	16.00	0.25	10.37	4.00	0.03
1	all isolates	0.37	0.76	0.04	0.008	4.79	5.72	4.75	5.82	0.05
2	all isolates	0.33	0.70	0.05	0.009	3.90	7.61	3.88	5.39	0.04
3	all isolates	0.29	0.70	0.05	0.007	3.73	9.18	4.17	6.06	0.04
4	all isolates	0.24	0.67	0.03	0.006	3.35	4.11	4.37	5.61	0.04
5	all isolates	0.21	0.59	0.02	0.006	2.57	2.62	4.74	4.97	0.04
1–5	all isolates	0.46	0.70	0.04	0.007	3.88	6.21	4.32	5.67	0.04

M, metronidazole; V, vancomycin; FDX, fidaxomicin; MIC, minimum inhibitory concentration; RIF, rifampicin; MXF, moxifloxacin; CLINDA, clindamycin; IMI, imipenem; CHLOR, chloramphenicol; TIG, tigecycline. MICs are reported as geometric means

Online Resource 5

Fidaxomicin MIC (mg/L) distributions for the most prevalent (n>30 isolates) ribotypes across all years of the study

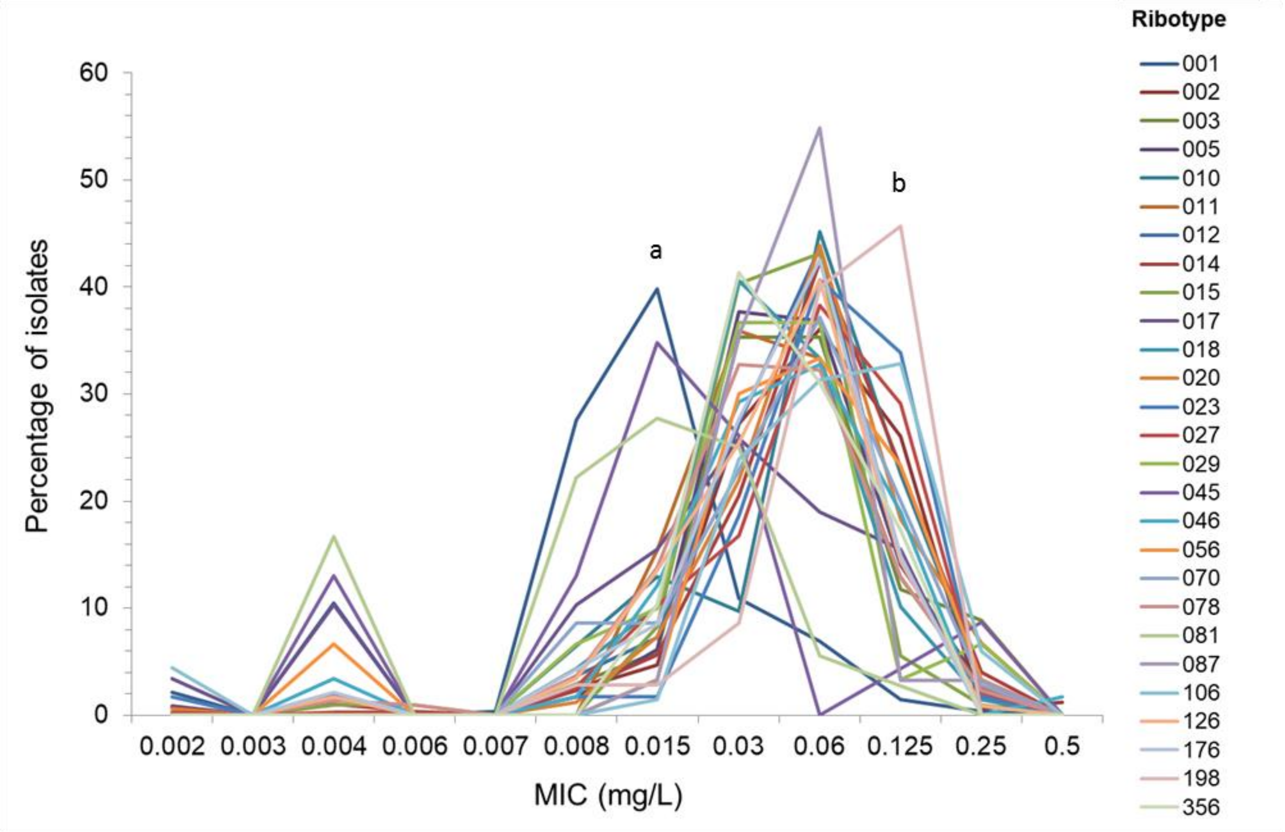

MIC, mimimal inhibitory concentration; RT, ribotype. [a]RT001 geometric mean MICs were significantly lower than the geometric mean fidaxomicin MIC for all ribotypes. [b]RT198 geometric mean MICs were significantly higher than the geometric mean fidaxomicin MICs for all ribotypes

Online Resource 6

MICs of the nine antimicrobials tested for all RT344 isolates

ID	M	V	FDX	RIF	MXF	CLINDA	IMI	CHLOR	TIG	Country
L,16.7569611	0.125	0.25	0.004	0.001	1	0.5	2	4	0.03	France
L,16.7570015	0.125	0.5	0.015	0.002	2	0.125	2	4	0.03	Slovenia
L,16.7570242	0.125	1	0.03	0.001	8	0.5	4	4	0.03	France
L,15.7104276	0.125	0.5	0.06	0.002	2	16	4	8	0.06	Greece
L,16.7569682	0.25	1	0.5	0.001	2	8	4	8	0.06	Belgium
L,16.7570132	0.125	1	>4	0.002	16	16	16	4	0.03	France

M, metronidazole; V, vancomycin; FDX, fidaxomicin; MIC, minimum inhibitory concentration; RIF, rifampicin; MXF, moxifloxacin; CLINDA, clindamycin; IMI, imipenem; CHLOR, chloramphenicol; TIG, tigecycline. MICs are reported as geometric means

Online Resource 7

Distribution of mean cumulative antimicrobial resistance scores for *Clostridium difficile* isolates across Europe in a) Year 1, b) Year 2, c) Year 3, d) Year 4 and e) Year 5 of the *Clos*ER study

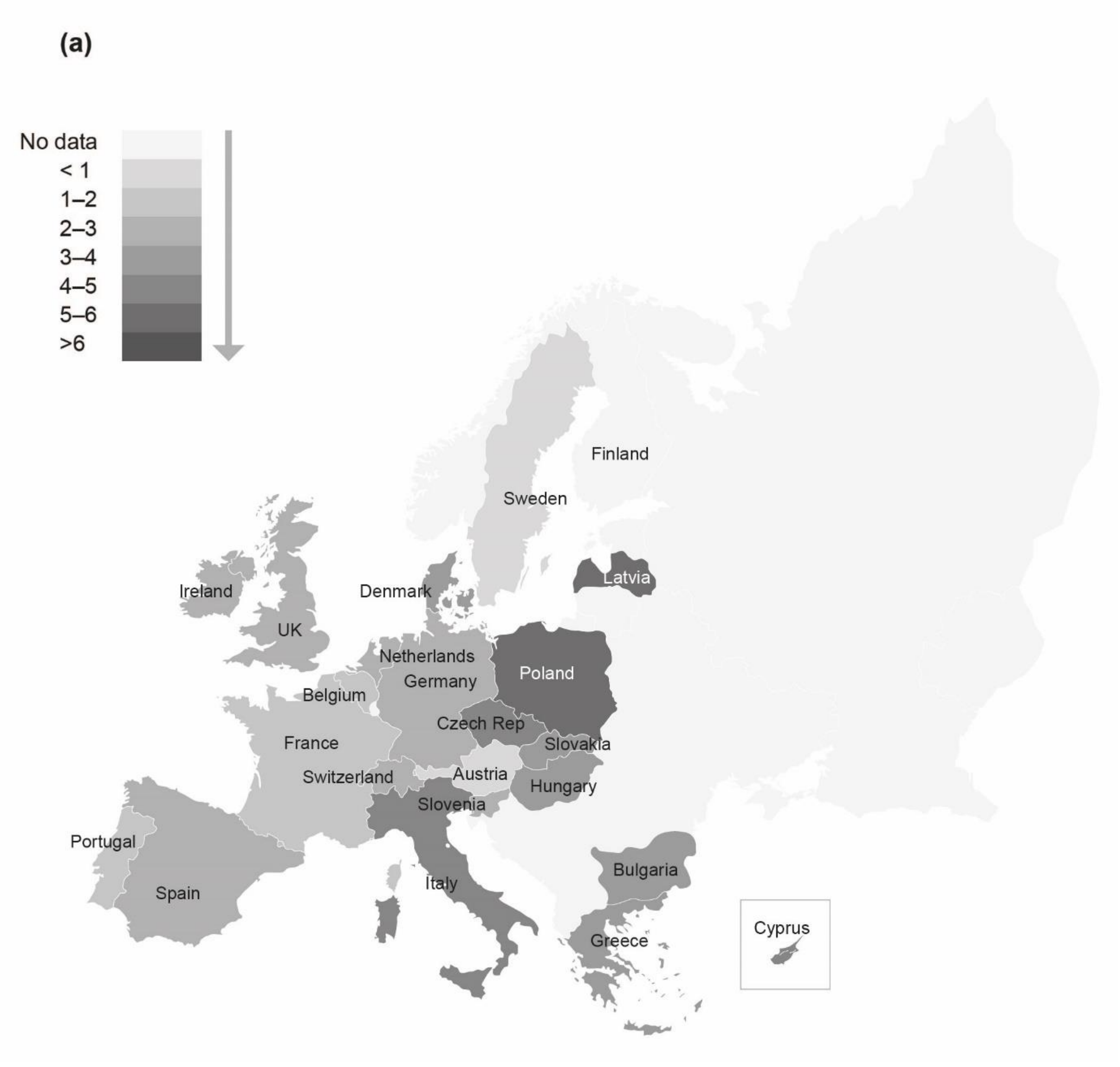

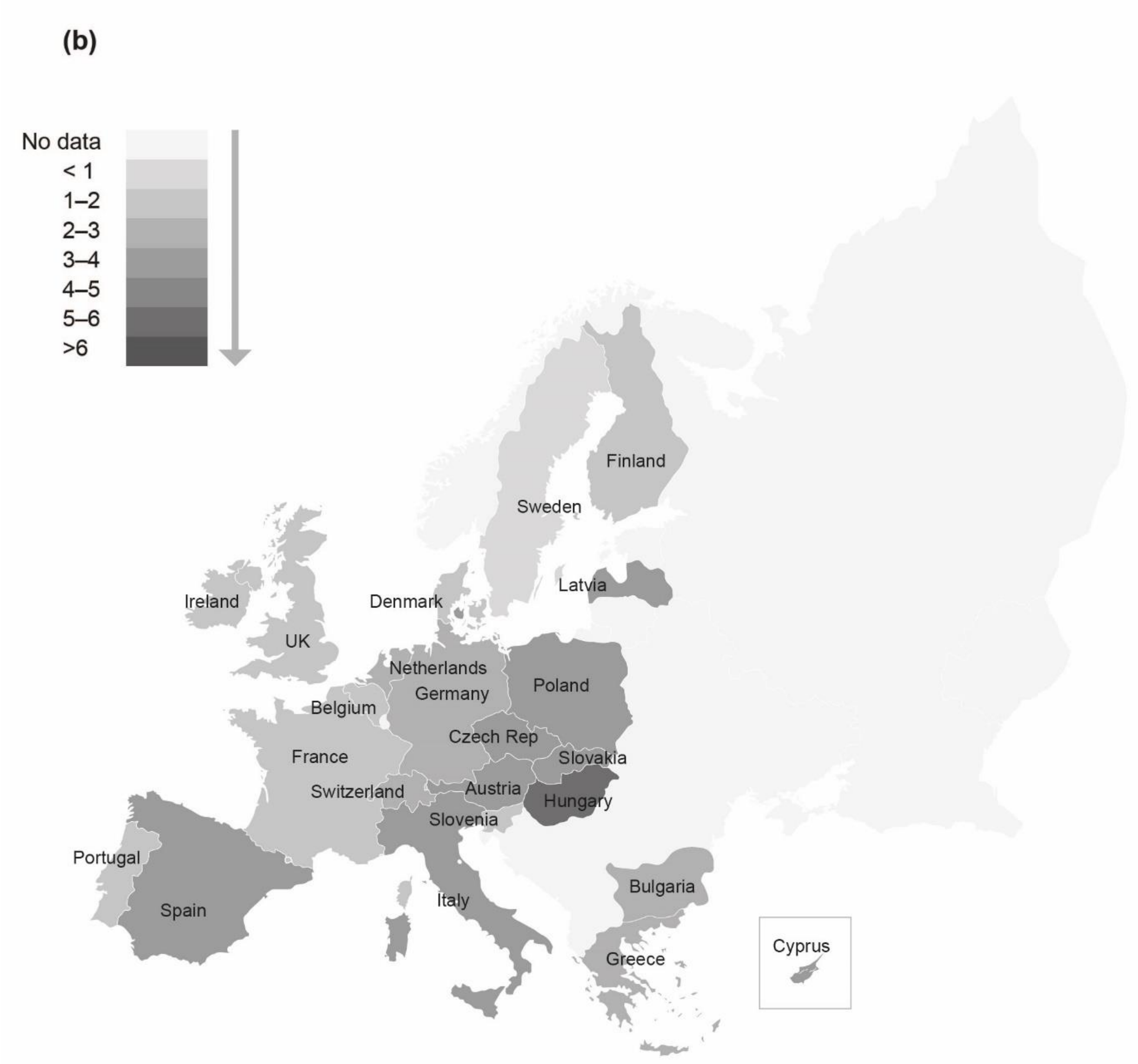
(b)
No data
< 1
1–2
2–3
3–4
4–5
5–6
>6
Finland
Sweden
Latvia
Ireland
Denmark
UK
Netherlands
Germany
Poland
Belgium
Czech Rep
France
Slovakia
Switzerland
Austria
Hungary
Slovenia
Portugal
Bulgaria
Italy
Spain
Cyprus
Greece

108

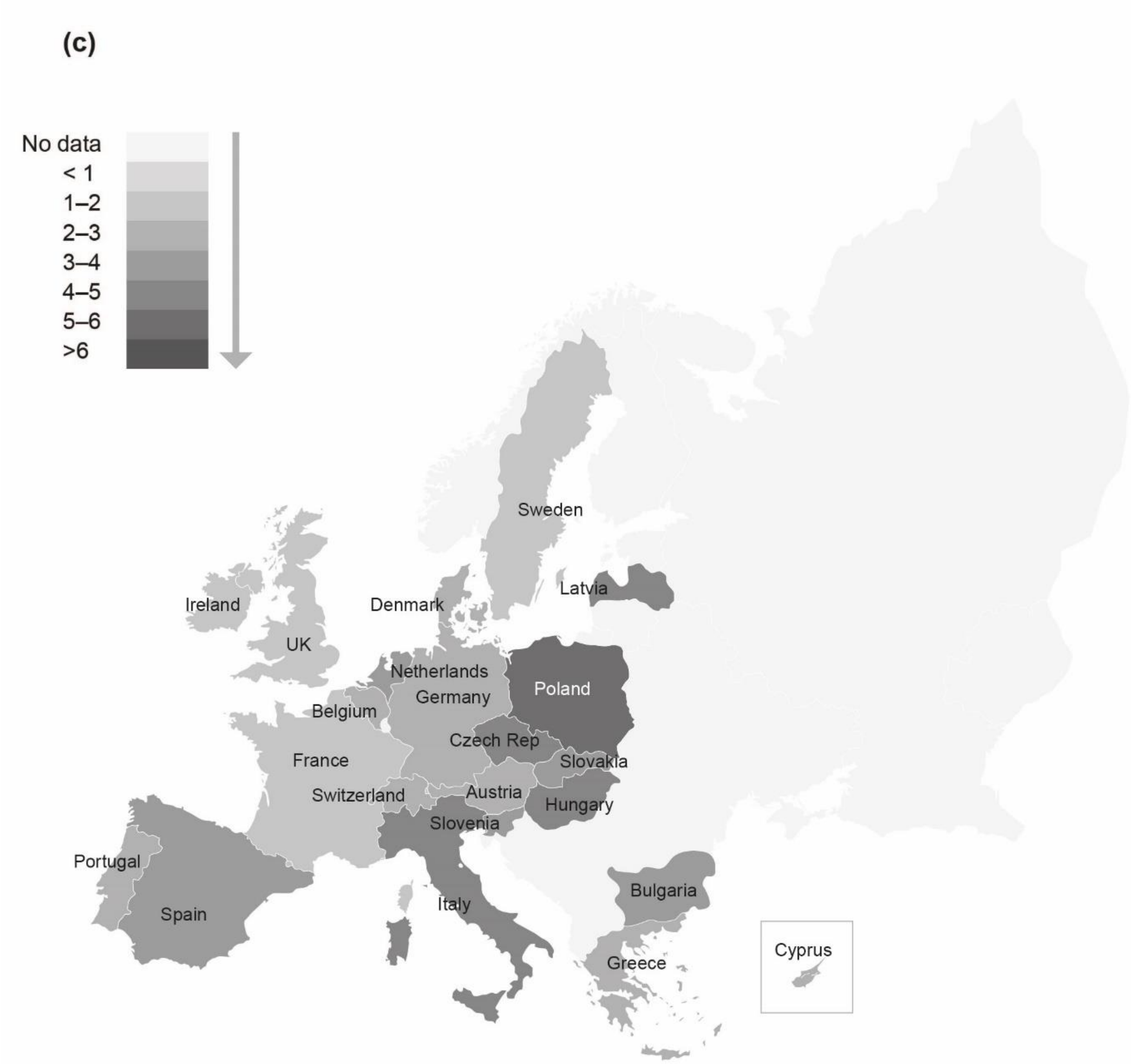
(c)
No data
< 1
1–2
2–3
3–4
4–5
5–6
>6
Sweden
Latvia
Ireland
Denmark
UK
Netherlands
Poland
Germany
Belgium
Czech Rep
France
Slovakia
Switzerland
Austria
Hungary
Slovenia
Portugal
Bulgaria
Italy
Spain
Cyprus
Greece

(d)

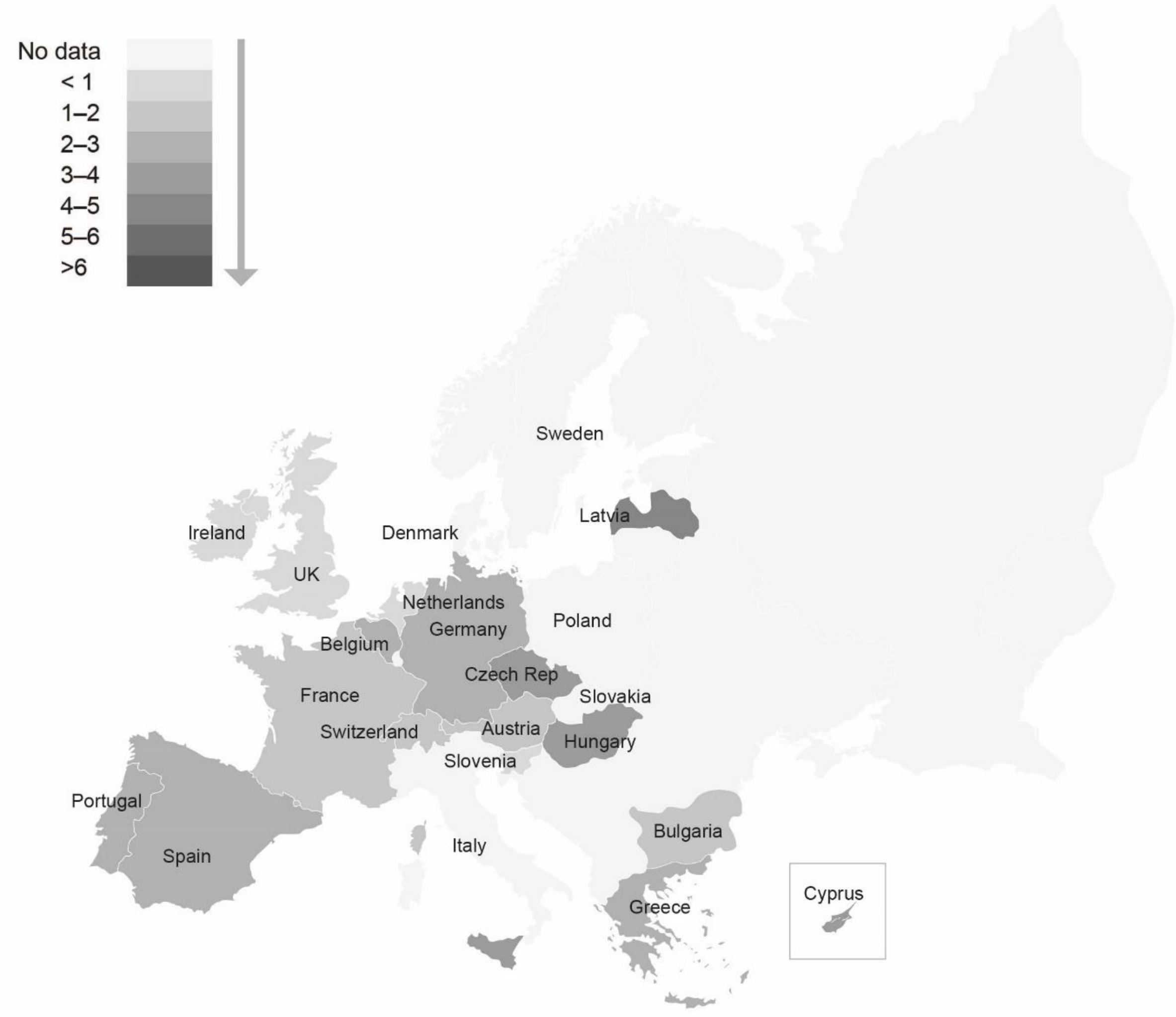
No data
< 1
1–2
2–3
3–4
4–5
5–6
>6
Sweden
Latvia
Ireland
Denmark
UK
Netherlands
Germany
Poland
Belgium
Czech Rep
France
Slovakia
Switzerland
Austria
Hungary
Slovenia
Portugal
Bulgaria
Italy
Spain
Cyprus
Greece

(e)

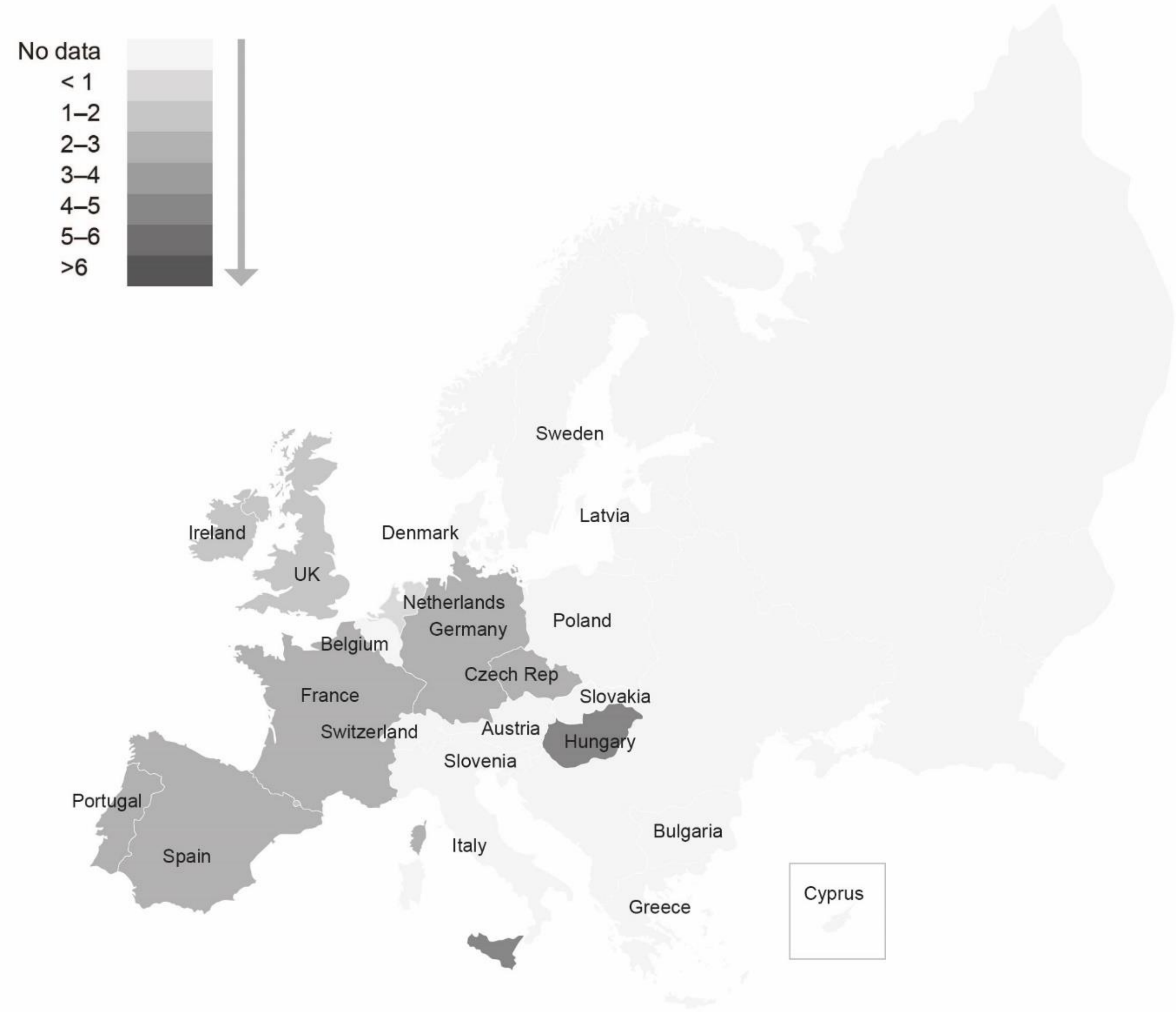
No data
< 1
1–2
2–3
3–4
4–5
5–6
>6
Sweden
Latvia
Ireland
Denmark
UK
Netherlands
Germany
Poland
Belgium
Czech Rep
France
Slovakia
Switzerland
Austria
Hungary
Slovenia
Portugal
Bulgaria
Italy
Spain
Cyprus
Greece

112

113

Online Resource 8

Cumulative resistance score versus ribotype diversity score for each country, Years 1–5 of the *Clos*ER study

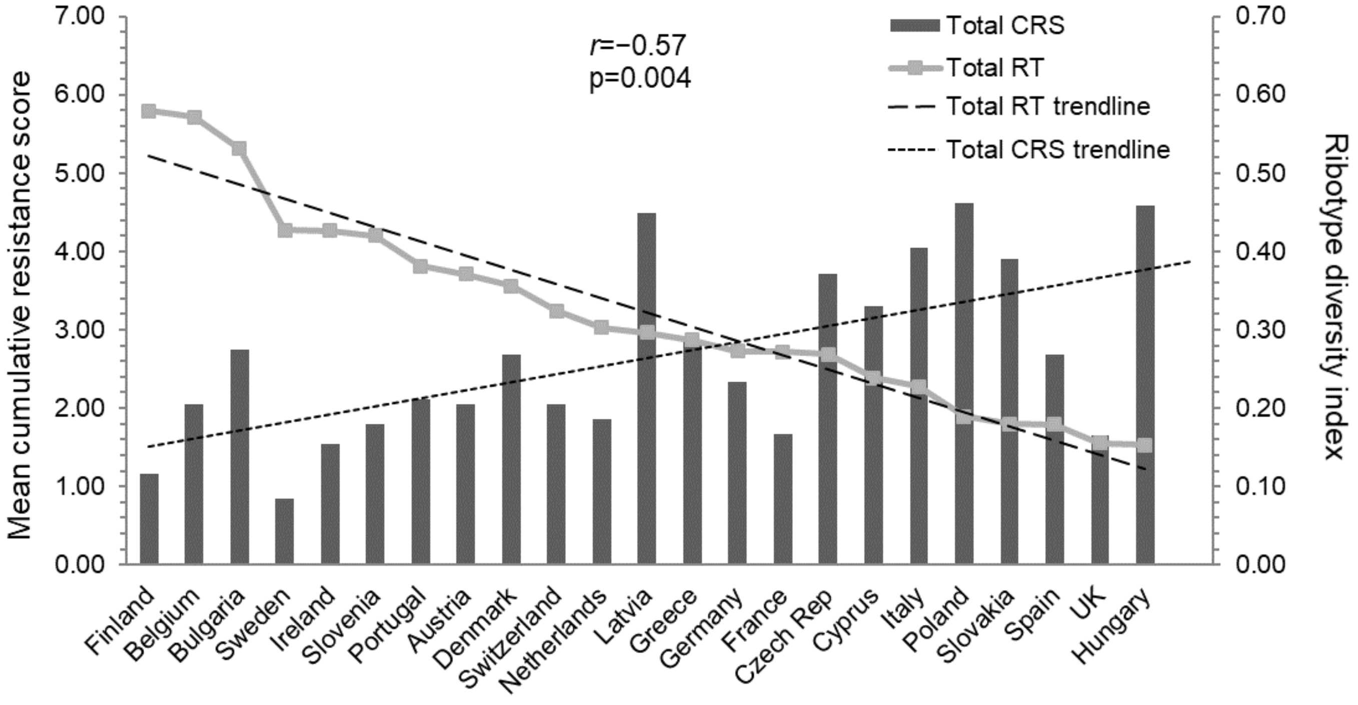

Supplement: Supplementary file 1 — (PDF 825 kb) [file 10096_2019_3708_MOESM1_ESM.pdf]
